# Electrospun fibre diameter and its effects on vascular smooth muscle cells

**DOI:** 10.1007/s10856-021-06605-8

**Published:** 2021-10-09

**Authors:** James Alexander Reid, Alison McDonald, Anthony Callanan

**Affiliations:** School of Engineering, The University of Edinburgh, Edinburgh, UK

## Abstract

Bypass grafting is a technique used in the treatment of vascular disease, which is currently the leading cause of mortality worldwide. While technology has moved forward over the years, synthetic grafts still show significantly lower rates of patency in small diameter bypass operations compared to the gold standard (autologous vessel grafts). Scaffold morphology plays an important role in vascular smooth muscle cell (VSMC) performance, with studies showing how fibre alignment and surface roughness can modulate phenotypic and genotypic changes. Herein, this study has looked at how the fibre diameter of electrospun polymer scaffolds can affect the performance of seeded VSMCs. Four different scaffolds were electrospun with increasing fibre sizes ranging from 0.75 to 6 µm. Culturing VSMCs on the smallest fibre diameter (0.75 µm) lead to a significant increase in cell viability after 12 days of culture. Furthermore, interesting trends were noted in the expression of two key phenotypic genes associated with mature smooth muscle cell contractility (myocardin and smooth muscle alpha-actin 1), whereby reducing the fibre diameter lead to relative upregulations compared to the larger fibre diameters. These results showed that the smallest (0.75 µm) fibre diameter may be best suited for the culture of VSMCs with the aim of increasing cell proliferation and aiding cell maturity.

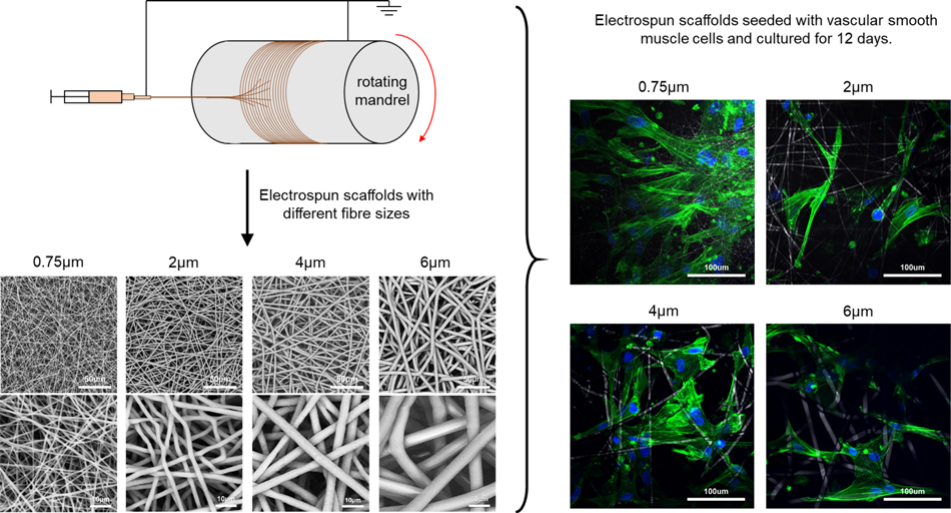

## Introduction

Cardiovascular disease is the leading cause of mortality worldwide, accounting for upwards of 23% of all deaths [1]. To treat this, techniques such as bypass grafting have been implemented to divert blood flow around an arterial blockage [2]. The current gold standard is to use one of the patients own vessels, such as the saphenous vein, however, in many instances this is not possible, therefore synthetic alternatives are required [2]. Unfortunately, synthetic grafts are associated with much lower rates of patency (degree of openness) compared to autologous vessel grafts in small diameter bypasses such as the coronary artery [2]. Therefore, there is a requirement to develop novel solutions in an attempt to bridge the gap between the use of synthetic grafts and autologous grafts.

Current scaffold based strategies used in vascular tissue engineering include the use of mechanical cues (topography) and biochemical cues (extracellular matrices and proteins) [3–9]. These cues can be incorporated into the scaffold to improve their performance as substrates for cell adhesion and proliferation. Specifically, electrospinning is an exciting avenue that allows for a wide range of scaffolds morphologies to be created [10–13]. This method has been used in many aspects of tissue engineering to mimic the structure of the native ECM, providing the cells with the correct mechanical cues [14]. Furthermore, work focussing on creating optimal environments for vascular smooth muscle cells (VSMCs) has shown that both scaffold morphology and pore size can have a drastic effect on the performance of VSMCs [11, 15–18]. In addition, a study by Noriega et al. using chondrocytes found that altering fibre diameter had the effect of increasing the expression of certain phenotypic genes [19]. These studies suggest that there is merit to studying the effect of electrospun fibre diameter on VSMC performance, with the aim of bridging the gap in material performance between synthetic grafts and autologous vessel grafts.

Herein, this study has looked at how the fibre diameter of electrospun polymer scaffolds can affect the performance of seeded VSMCs. We believe that altering scaffold fibre diameter will lead to different levels of cellular infiltration and should lead to phenotypic and genotypic alterations in the seeded VSMCs. It is our aim that this study will add to the understanding of what scaffold morphology is best suited to VSMCs.

## Methods and materials

### Electrospinning

Polycaprolactone (PCL) was either dissolved into Hexafluoropropane (HFIP); a 5:1 mixture of Chlorofrom:Methanol (C:M); or a 10:1 mixture of C:M at varying concentrations to achieve four different solutions (Table 1). Briefly, the electrospun fibres were spun as one continuous fibre onto an aluminium foil covered 8 cm diameter rotating mandrel in an environment that consisted of 18–24 °C and 40/60% relative humidity. A rotating collector was used to give a more even distribution of the fibres with better control over thickness. Note that there was no tubularity in the scaffolds from the rotating collector after being punched. The electrospinning parameters used to spin each set of fibres can be seen in Table 1.

**Table 1:** Electrospinning parameters used to manufacture four scaffolds with different fibre diameters

Fibre diameter (µm)	Polymer concentration (%)	Solvent used	Needle bore (mm)	Flow rate (mL/h)	Total volume (mL)	Distance between needle tip and mandrel (cm)	Positive voltage (kV)	Negative voltage (kV)	Mandrel rotational speed (rpm)
0.77 ± 0.14	8	HFIP	0.4	1.2	8	12	+12	0	250
2.06 ± 0.26	12	HFIP	0.4	1.7	8.5	15	+12	0	250
3.91 ± 0.40	20	5:1 C:M	0.8	5	18	23	+15	−4	250
5.91 ± 0.56	20	10:1 C:M	0.8	6	18	21	+23.5	0	250

### Scanning electron microscopy

Scaffolds were imaged using a Hitachi TM4000 tabletop Scanning electron microscope (SEM). No sputter coating was required.

### Fibre and pore measurements

SEM images were imported directly into ImageJ and analysed. Images were thresholded and then analysed using the DiameterJ plugin [20].

### Mechanical characterisation

Tensile scaffold properties were measured using an Instron 3367 testing rig. Briefly, scaffolds were cut into 40 × 5 mm strips for analysis with measurements performed on a starting gauge length of 20 mm. Scaffolds were stretched until failure at 10 mm/min. Incremental Young’s Moduli were then calculated using a previously described method [21].

### Contact angle measurement

Contact angle was measured on each scaffold using a DMK 41AU02 monochrome camera at a frequency of 5 Hz. Briefly, a 5 µl droplet of water was placed on the scaffold whilst images were being taken. Analysis was performed on ImageJ using the LBADSA plugin [22].

### Scaffold porosity

Scaffold porosity (%) was calculated using the density of PCL, the weight of the scaffold and volume of the scaffold. The thickness of the scaffold was measured in order to calculate the volume of the scaffold. This was done using a DMK 41AU02 monochrome camera. All scaffolds were punched out using a 10 mm diameter punch.

### Cell culture and scaffold seeding

Human umbilical vein smooth muscle cells (HUVSMCs) were expanded to passage 4 in a 5% CO_2_/37 °C atmosphere. HUVSMCs were expanded using smooth muscle cell growth medium (Sigma-Aldrich) and then cultured in these experiments using DMEM supplemented with 10% v/v FBS; 1% v/v penicillin/streptomycin; and 1% v/v non-essential amino acids. HUVSMCs were lifted for scaffold seeding at 80% confluence. Briefly, 10 mm diameter scaffolds were punched out and sterilized in 70% ethanol before being soaked in basal medium overnight (non-supplemented DMEM). Scaffolds were then seeded in a 48-well plate. 25,500 cells/cm^2^ were drip seeded in 20 µl of culture medium (supplemented DMEM) onto the middle of the scaffold. After 30 min a further 30 µl of medium was added to stop the cells from drying out. After a further 30 min, medium in each well was topped up to 500 µl. Medium was subsequently replaced every 48 h.

### Cell viability

Cell viability was measured using the CellTiter-Blue^®^ cell viability assay at 1, 6 and 12 days as per manufacturer’s instructions (Promega). Briefly, cell seeded scaffolds were removed and placed in new wells. Each well was topped up with a 4:1 ratio of media and CellTiter-Blue assay. The plate was lightly shaken for 1 min and then wrapped in aluminium foil and incubated for 3.5 h. After incubation, 100 μL samples (x3) of the media/assay were taken from each scaffold/well and pipetted into a black well plate. The plate was measured in a Modulus™ II microplate reader at excitation: 525 nm and emission: 580–640 nm.

### Cell staining

Scaffolds used for cell staining were washed thrice in phosphate buffer saline (PBS) and fixed in 10% v/v formalin solution in PBS overnight. Cells were permeabilized in 0.2% v/v TritonX-100 solution then stained in 0.1% v/v 1000X Phalloidin-iFluor™514 conjugate (F-actin) and in DAPI (cell nuclei). Fluorescent images were taken using a bespoke coherent anti-stokes Raman (CARS) system. Z-stack images were taken after 12 days of culture to assess the amount of cell infiltration on each scaffold.

### 2Cell infiltration measurements

Cellular infiltration was measured using the DAPI and phalloidin stained Z-stack images on ImageJ (NIH) whereby the depth of cell intravasation was measured.

### Reverse transcription quantitative polymerase chain reaction (RT-qPCR)

RNA was extracted from the cell seeded scaffolds using a Tri-Reagent method and purified using Qiagen’s RNeasy spin column system. Real-time polymerase chain reaction was performed using a LightCycler^®^ 480 Instrument II and Sensifast™ SYBR^®^ High-ROX system. Forward and reverse sequences were either designed or used from literature and are displayed in Table 2. Relative quantification of RT-PCR results was carried out using the 2^−ΔΔ*ct*^ method [23]. Gene expression levels were expressed relative to GAPDH (housekeeping gene) and normalised to 70% confluent HUVSMCs on tissue culture plastic.

**Table 2:** Primer sequences used in RT-qPCR analysis

Gene	Primer	Sequence	Reference
Glyceraldehyde 3-phosphate dehydrogenase	GAPDH (forward)	GTCTCCTCTGACTTCAACAG	[56]
	GAPDH (reverse)	GTTGTCATACCAGGAAATGAG	
Smooth muscle actin alpha 1	Α-actin1 (forward)	CCGACCGAATGCAGAAGGA	[28]
	Α-actin1 (reverse)	ACAGAGTATTTGCGCTCCGAA	
Myocardin	Myocardin (forward)	GGGTCTGAGCATTCCTTGCT	Self-designed
	Myocardin (reverse)	CTGGACGTTTCAGTGGTGGT	

### Statistical analysis

Data was expressed as mean ±1 standard deviation. Statistical analysis was performed using one-way ANOVA with post-hoc Tukey test.

## Results

### Scaffold properties

**Fig. 1:**
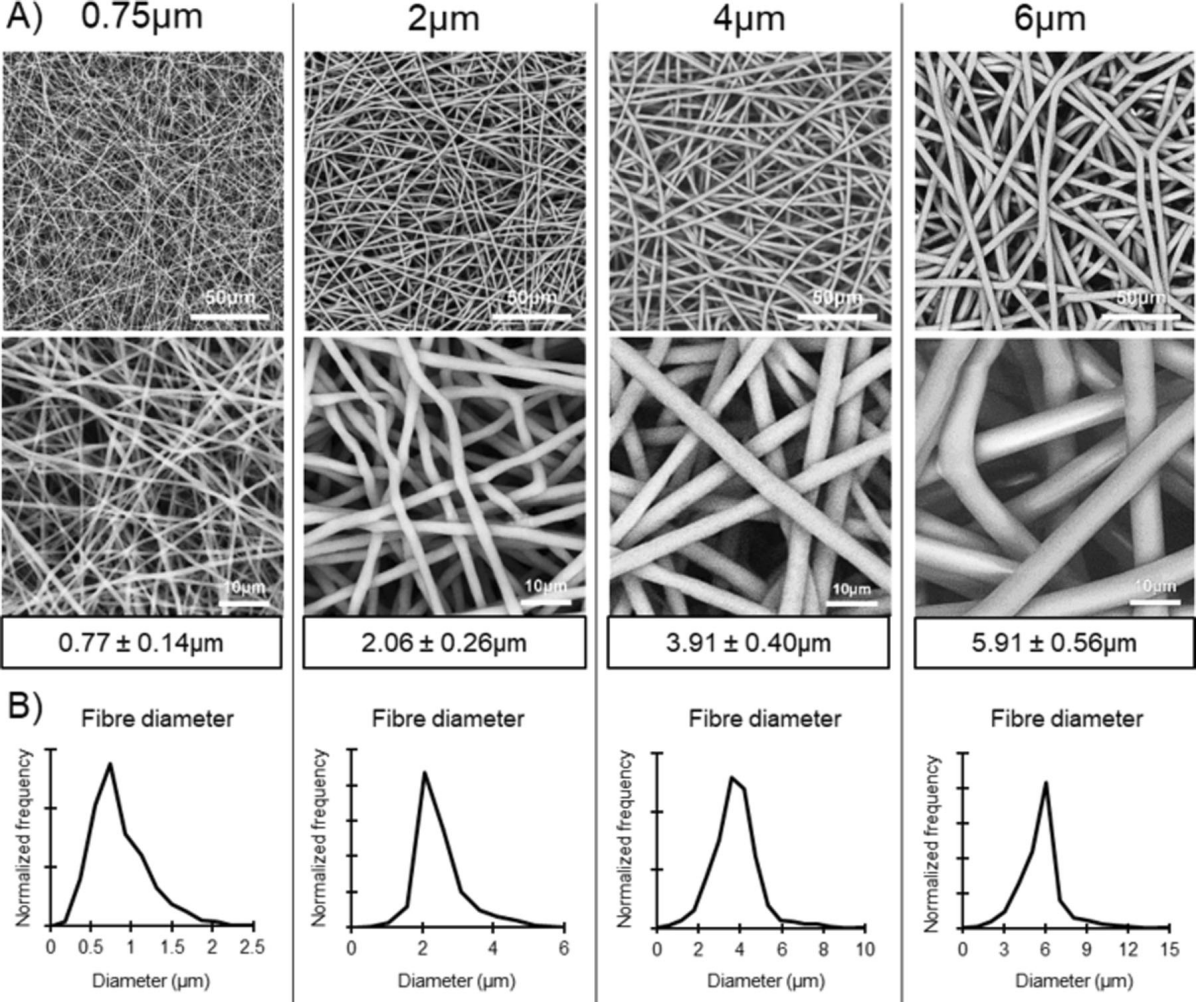
**A** SEM images of the four electrospun scaffolds with increasing fibre diameters. **B** Normalized frequency of fibre diameters found in each scaffold

Four different fibre morphologies were achieved by altering the electrospinning parameters. Firstly, increasing fibre diameters of 0.77 ± 0.14, 2.06 ± 0.26, 3.91 ± 0.40 and 5.91 ± 0.56 µm were noted for the four scaffolds (*p* < 0.001 between each group), as seen in Fig. 1. In addition, the fibre alignment of each scaffold appears fairly random and displays no alignment in a particular direction. This can be seen in the SEM images. For simplicity, these four scaffolds will be referred to as 0.75, 2, 4 and 6 µm for the rest of this manuscript.

**Fig. 2:**
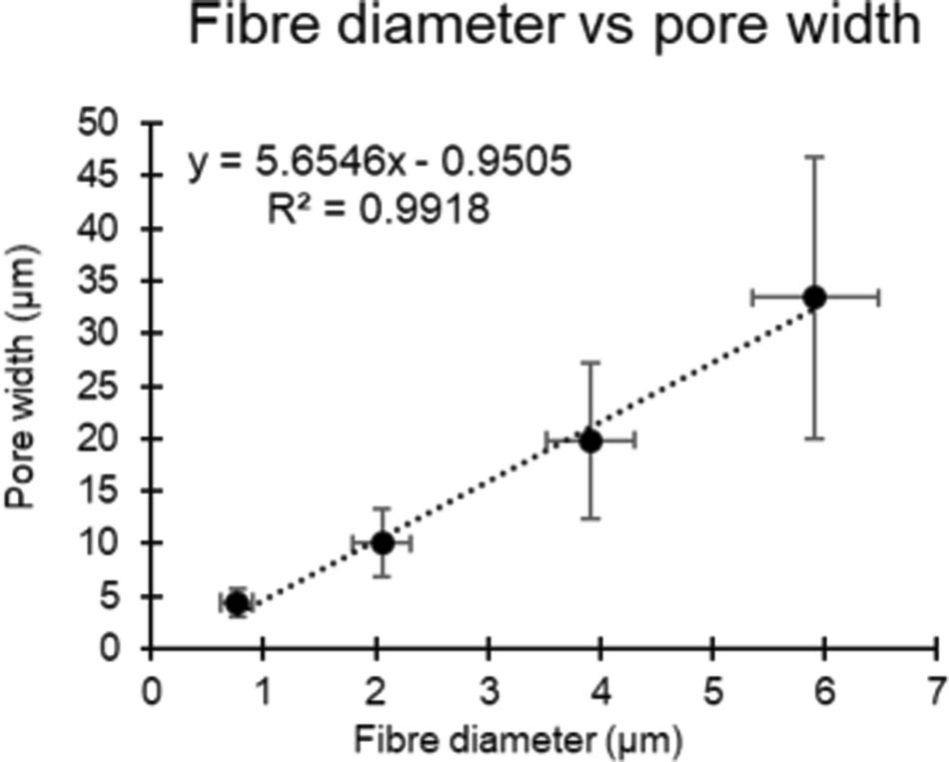
Correlation between fibre diameter and pore width

Furthermore, a very strong correlation between fibre diameter and pore width was noted for all four scaffold morphologies, with an *R*^2^ value of 0.9918. Pore widths ranged from 4.37 ± 1.29 μm for the smallest (0.75 µm) fibre diameter up to 33.41 ± 13.37 µm for the largest (6 µm) fibre diameter. These correlations can be seen in Fig. 2.

### Scaffold mechanical properties

Firstly, it was noted that the 2 µm scaffold morphology had the highest ultimate tensile strength (UTS), highest failure strain and the highest stiffness at all 5 strain bands measured (Table 3). The UTS for these scaffolds dropped either side of the 2 µm fibre diameter with the 2 µm fibre diameter showing a significantly higher UTS (*p* < 0.012 for all 3 comparisons). While the failure strain was higher in the 2 µm fibre diameter compared to the three other morphologies, it was only significantly higher than the 0.75 µm fibre diameter (*p* = 0.000 for the 0.75 µm scaffold, *p* = 0.164 and 0.168 for the 4 µm scaffold and 6 µm scaffold, respectively). Furthermore, the 0.75 µm not only had a significantly lower failure strain than the 2 µm fibre diameter, as previously mentioned, it also had significantly lower failure strains than the 4 and 6 µm fibre diameters (*p* = 0.011 for both). There was no notable or significant difference in contact angle across all the scaffolds groups, with a variation between 125.6 to 138.1°. These values can all be seen in Table 3.

**Table 3:** Physical and mechanical properties of all four scaffolds

Scaffold Name	Fibre diameter (µm)	Pore diameter (μm)	Ultimate tensile strength (mpa)	Failure strain (%)	Contact angle at 0.2 s (°)	Stiffness (MPa) at given strain bands				
						0–1 %	1–2 %	2–3 %	3–4 %	4–5 %
0.75 µm	0.77 ± 0.14	4.3 ± 1.3	1.41 ± 0.09	357 ± 79	130.7 ± 0.9	3.77 ± 1.75	6.49 ± 1.64	7.35 ± 0.66	7.09 ± 0.64	6.31 ± 0.67
2 µm	2.06 ± 0.26	10.1 ± 3.2	1.98 ± 0.31	1090 ± 283	130.6 ± 5.0	5.77 ± 0.53	9.04 ± 1.62	8.76 ± 1.78	7.88 ± 1.77	6.76 ± 1.56
4 µm	3.91 ± 0.40	19.8 ± 7.4	1.45 ± 0.21	819 ± 117	134.2 ± 3.9	3.51 ± 0.55	5.97 ± 0.25	6.05 ± 2.05	5.64 ± 0.25	4.77 ± 0.62
6 µm	5.91 ± 0.56	33.4 ± 13.4	1.22 ± 0.07	821 ± 127	130.1 ± 2.2	2.07 ± 0.16	3.53 ± 0.39	3.63 ± 0.43	3.43 ± 0.53	3.14 ± 0.39

Likewise, the stiffness of each scaffold at the five different strain bands followed a similar pattern. The 2 µm fibre diameter scaffold showed a higher stiffness at all five strain bands (see Table 3) compared to the three other fibre diameters, and the 6 µm fibre diameter showed a lower stiffness at all five strain bands. Significance was noted at all 5 strain bands between the 2 µm fibre diameter and the 4 and 6 µm fibre diameters (*p* < 0.05 in all cases) and in some cases significance between the 2 µm fibre and small 0.75 µm fibre diameters (*p* = 0.053 for the 0–1% strain band; *p* = 0.042 for the 1–2% strain band).

### Cell imaging

**Fig. 3:**
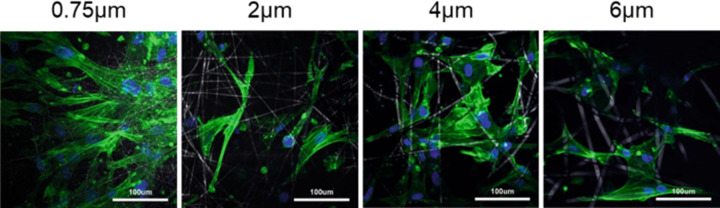
2D confocal fluorescent images of nuclei (DAPI—blue) and Actin (phalloidin—green) stained HUVSCMs on all four scaffold morphologies

2D confocal fluorescence imaging (Fig. 3) showed that a layer of SMCs were formed on the 0.75 µm fibre scaffold after 12 days of culture showing the phenotypic characteristics of healthy SMCs. Furthermore, while no clear monolayer could be noted on the three other morphologies, they were all able to maintain the elongated phenotypic characteristics of SMCs like the small fibre diameter scaffold.

**Fig. 4:**
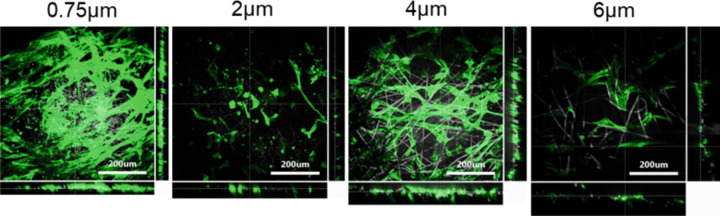
Z-stack confocal fluorescence images of actin stained HUVSMCs after 12 days of culture on all four scaffold morphologies. These images were used to deduce the quantity of cell infiltration

Z-stacks of Actin stained SMCs showed how increasing fibre diameter had the effect of increasing cell infiltration into the scaffold. Cell infiltrations of ~40, 42, 61 and 82 µm were noted for the four scaffolds, as seen in Fig. 4.

### Cell viability

**Fig. 5:**
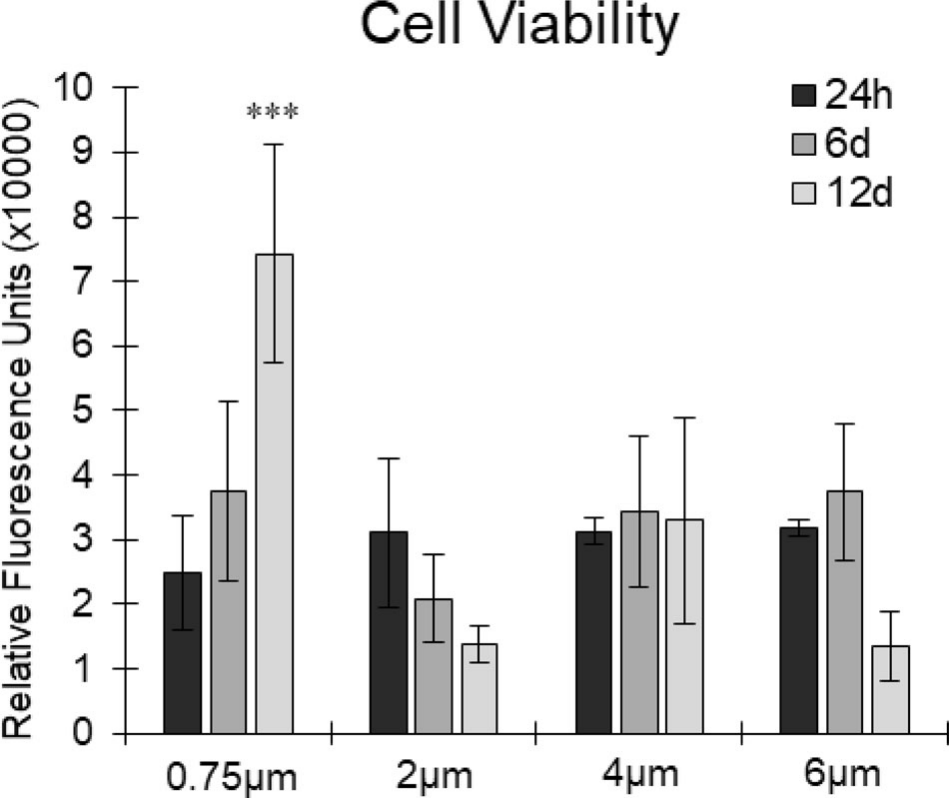
Cell viability of HUVSMCs on all four scaffolds after 1, 6 and 12 days of culture. *n* = 4, error bars = SD, ***p < 0.001

Cell viability was measured using a CellTiter-Blue^®^ Cell Viability Assay and showed some interesting results, as seen in Fig. 5. First, cell viability in the 0.75 µm fibre diameter scaffold after 12 days of culture was significantly higher than in the three other morphologies, suggesting that a smaller fibre morphology may be more suited for the growth of SMCs. On the contrary, the 2 µm fibre diameter and 6 µm fibre diameter both had reductions in cell viability, whereas the 4 µm fibre diameter showed no real change in cell viability over the 12 days of culture.

### RT-qPCR

**Fig. 6:**
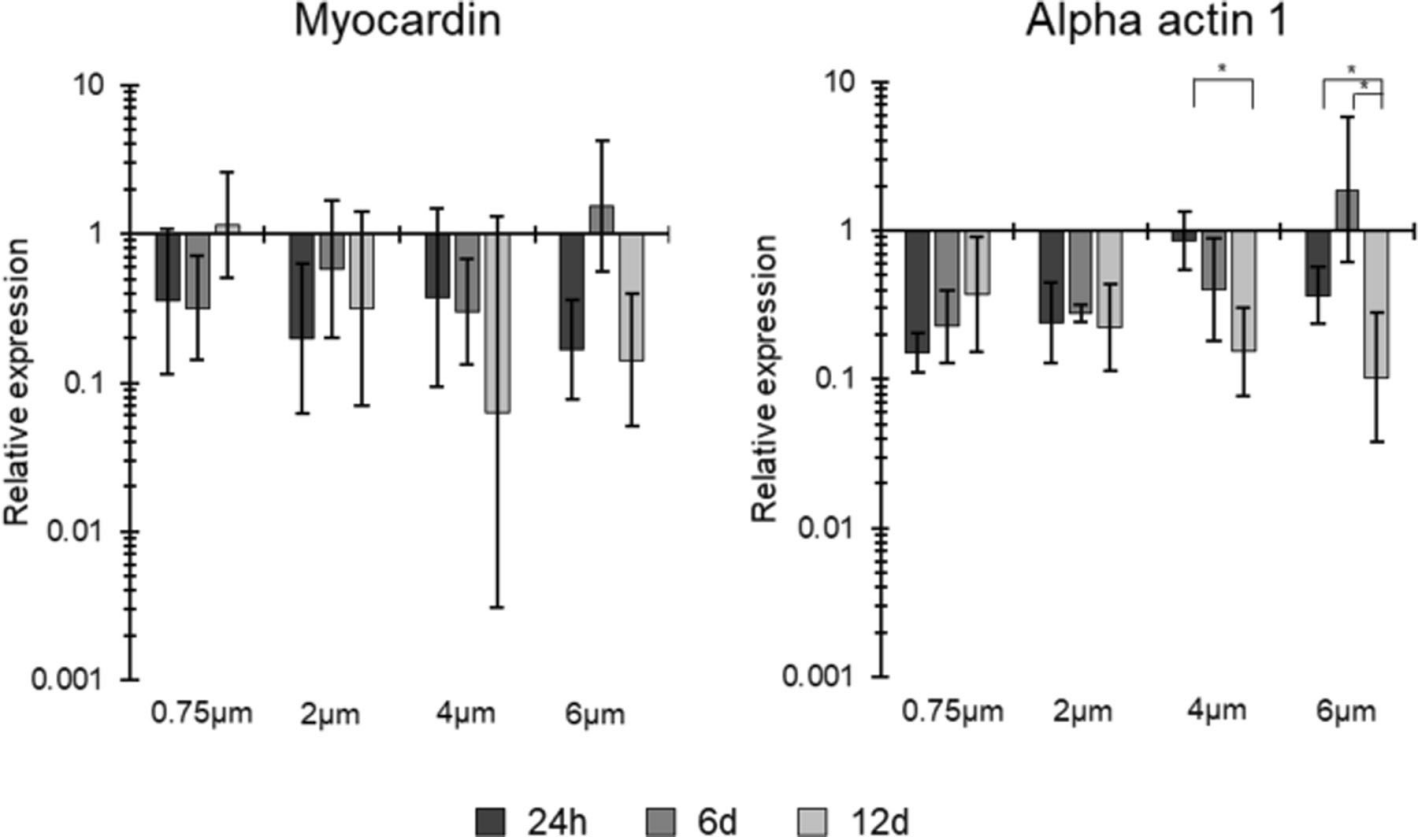
RT-qPCR results for myocardin and alpha actin 1 on all four scaffolds after 1, 6 and 12 days of culture. All results were normalised to 70% confluent HUVSMCs on tissue culture plastic. *n* ≥ 3, error bars = SD. *p < 0.05

RT-PCR results showed some interesting trends in VSMC gene expression between the four scaffold morphologies over the 12 days of culture, as seen in Fig. 6. Myocardin is a key phenotypic gene in smooth muscle cells and is heavily involved in their contractile functionality, with upregulations being associated with cell maturity [24–27]. In this study, the 0.75 µm fibre diameter led to an upregulation in myocardin between 6 and 12 days of culture, whereas in the three larger fibre diameters, a downregulation was noted between the same two timepoints. Likewise, smooth muscle alpha-actin 1 is associated with maturing smooth muscle cells and their contractile functionality [28]. This study found an upregulation of smooth muscle alpha-actin 1 on the 0.75 µm fibre between 24 h and 12 days of culture. On the contrary, all three other scaffolds showed a downregulation. The only significance noted was in the alpha-actin 1 results, whereby significant downregulations were noted in the 4 and 6 µm fibre diameters between 24 h and 12 days of culture.

## Discussion

In this study, PCL was used as the polymer for electrospinning. PCL is a widely used polymer in tissue engineering applications due to its versatility and its FDA approval for clinical use [29, 30]. It has been extensively used to manufacture different types of scaffolds, especially electrospun fibrous scaffolds which require a polymer that can be easily used with different solvents [31–34]. Electrospinning is a flexible scaffold manufacturing technique that allows for a range of different morphologies to be created [10]. This study manufactured four different scaffolds with increasing fibre diameters ranging from 0.77 ± 0.14 µm to 5.91 ± 0.56 µm with relatively equal increments between each scaffold (Fig. 1). Interestingly, a very strong correlation was noted between the fibre diameter and the average pore width found on all four scaffolds (*R*^2^ = 0.9918) (Fig. 2). This allowed for the study of fibre diameter/pore width and cellular infiltration and their effects on seeded SMCs.

While the approaches used in this study were optimised to create certain fibre sizes and morphology, it is poignant to mention the effect of some of the variable parameters in the fibre mat formation. We have used different solvents combinations, voltages, polymer concentrations, needle bore size and distance to modify the specific sizes of the fibres. These parameter changes in particular solvent differences have been linked to modifications in crystallinity, melting temperature and surface roughness [35]. In addition, it has been reported that the mechanical properties of electrospun fibres are influenced by crystallinity and molecular orientation, and their crystal structure is controlled by the processing parameters, which can include the electrical and rheological properties [36, 37]. While we did not look at crystallinity effects, based on our SEM analysis we can confirm no adverse effects on fibre roughness. Interestingly, this approach of varying multiple parameters to achieve a range of fibre sizes has been commonly done in the literature [11, 38, 39]. One notable study showed that the best results were achieved using a multi-parameter approach to achieve a range of fibre sizes [38].

As expected, increasing fibre diameter and pore width led to an increase in cell infiltration depth. Depths of ~40, 42, 61 and 82 µm were noted for the 0.75, 2, 4 and 6 µm scaffolds, respectively (Fig. 4). This phenomenon is expected and has been seen in previous studies where increasing the pore size of a scaffold has led to increased cellular infiltration [40, 41]. Phipps et al. noted a six to sevenfold increase in mean cellular infiltration of Mesenchymal Stem cells when average pore area was increased from ~400 µm^2^ to 1800 µm^2^ [41]. Likewise, Whited et al. found that increasing pore diameter from less than 10 to 89 µm lead to significant increases in osteoblasts infiltrating to depths greater than 600 µm after 6 days of culture [40]. While these cell types are different to the ones used in this study, the same principle applies whereby a larger pore size offers more space for cell infiltration.

Fluorescence images of DAPI and phalloidin stained cells showed a monolayer of SMCs on the 0.75 and 4 µm fibre diameter scaffolds (Figs. 3, 4). In contrast, the 6 µm scaffold appears to show cells infiltrating into pores and growing into the scaffold. While the 2 µm fibre diameter scaffold may not have a full monolayer of cells growing, it does show cells growing along a singular plane in the scaffold, which suggests that no infiltration is occurring. In vivo, VSMCs adopt an elongated morphology and are found interwoven around the ECM in thick layers of 40–60 cells [42]. This would suggest that the 6 µm scaffold would be optimal for the growth of VSMCs. However, the results presented in this study suggest that the 0.75 µm fibre scaffold led to increased cell performance. VSMCs tend to grow in very compact formations in vivo and therefore the 6 µm fibre scaffold may not have allowed for adequate cell-cell interactions due to the increase in space on offer for cell growth. This in turn may have decreased the amount of paracrine communication between the cells, reducing their proliferative performance. Studies have shown that decreased cell-cell communication in seeded scaffolds can lead to reduced cell growth and altered phenotypic gene expression in various cell types [43, 44].

The 0.75 µm fibre scaffold led to the formation of VSMC bundles, whereby the cells realigned themselves and adopted a compact morphology commonly seen in in vitro 2D cultures [45, 46]. Furthermore, the 0.75 µm scaffold also showed significantly higher cell viability (Fig. 5) after 12 days of culture compared to the three other morphologies. While this shows that the 0.75 µm scaffold is well suited for VSMCs culture in vitro and leads to increased cell proliferation, it does not necessarily mean that this scaffold is optimal for the long-term 3D culture of VSMCs. Work by Bono et al. found that culturing human umbilical artery SMCs in a 3D micro-environment (collagen hydrogel) led to increased cell alignment compared to 2D culture on tissue culture plastic [47]. Interestingly, they noted short term (5 days) reduction in the expression of calponin (a phenotypic gene associated with contractility) in the 3D culture compared to the 2D culture. They ascribed this reduction to the cells adapting to the 3D micro-environment causing temporal variation in cell-matrix interaction [48]. Similarly, Lin et al. found that human induced pluripotent stem cell differentiated VSMCs downregulated alpha smooth muscle actin and calponin when cultured in a 3D micro-environment compared to conventional 2D culture [49]. These results are similar to those found in the present study, whereby two phenotypic genes (alpha smooth muscle actin and myocardin) were downregulated in the 6 µm scaffold, possibly due to the same phenomena where increased infiltration means an increased amount of cell adaptation to the 3D micro-environment.

A relative upregulation of myocardin was noted in the 0.75 µm fibre diameter scaffold compared to the three larger fibre diameter scaffolds between 6 and 12 days of culture. Myocardin is a major phenotypic gene present in SMCs and is responsible for their contractile functionality [24–27]. As SMCs proliferate and mature, an upregulation of myocardin would be expected. A study by Raphel et al. showed that myocardin overexpression in vitro through the use of adenoviruses led to human embryonic stem cell differentiation into functional phenotypic mature smooth muscle cells [50]. Furthermore, smooth muscle alpha-actin 1 is associated with maturing smooth muscle cells and their contractile functionality [28]. Work by Sandbo et al. found that a downregulation of smooth muscle alpha-actin 1 led to a decrease in SMC migration [51]. This study found that the 0.75 µm fibre diameter increased the expression of this gene over a 12 day culture period, whereas the three other scaffolds lead to decreases in the expression of this gene over 12 days. These results would suggest that the 0.75 µm fibre diameter scaffold helped facilitate SMC maturation and improved their phenotypic functionality compared to the three larger fibre diameters. However, as previously mentioned, this downregulation may be due to the short-term cell adaptation to the 3D microenvironment found in the larger fibre diameters [48, 49].

There has been a plethora of work looking at how fibre alignment and other morphological aspects such as surface roughness affect SMCs. For example, work by Bashur et al. found that culturing rat aortic SMCs on aligned fibres led to increased cell alignment and promoted a synthetic phenotype when compared to culturing on randomly orientated fibres [52]. Likewise, Nivison-Smith et al. showed that increasing fibre alignment lead to a significant increase in the alignment of seeded primary human coronary artery SMCs after 24 h, 72 h and 120 h [53]. These results show that the effect of fibre alignment spans into long term culture, whereby even after the cells have started to proliferate, they are still evidently sensing the alignment of the scaffold. Similarly, work by Ng et al. showed that culturing human aorta SMCs on aligned PCL fibres increased cell proliferation and significantly increased the expression of alpha-smooth muscle actin and smooth muscle myosin heavy chain; two phenotypic genes that are associated with SMC contractility [54]. Furthermore, Zhou et al. showed that human umbilical artery SMCs are not only affected by fibre alignment, but also by the surface roughness of the fibre [55]. Increasing surface roughness had the effect of increasing both cell adhesion and cell proliferation, as well as increasing nuclei elongation. These results combined with the results presented in this present study demonstrate how SMCs are affected by their substrate’s morphology in several different ways.

## Conclusions

In conclusion, four different electrospun scaffolds with increasing fibre diameters ranging from 0.75 to 6 µm were electrospun. The largest (6 µm) fibre diameter led to increased cell infiltration compared to the three other morphologies. However, the smallest (0.75 µm) diameter fibre led to a monolayer of HUVSMCs growing, showing significantly higher cell viability after 12 days of culture compared to the three other morphologies. Furthermore, altering the fibre diameter had the effect of changing the levels of gene expression from the HUVSMCs. Most notably, the cells seeded onto the 0.75 µm fibre had elevated expressions of myocardin and smooth muscle actin-α 1 (two genes associated with mature SMCs) compared to the three other morphologies. These results show how altering the fibre diameter of the scaffold has clear effects on the seeded HUVSMCs. Our findings suggest that the 0.75 µm fibre diameter is most suitable for the seeding of SMCs with the aim of aiding cell maturity.

## Data Availability

Data available on request from the authors.
